# Confirm your subscription to continue receiving our Table of contents

**DOI:** 10.2807/1560-7917.ES.2025.30.13.250403m

**Published:** 2025-04-03

**Authors:** Eurosurveillance editorial team

**Affiliations:** 1 European Centre for Disease Prevention and Control (ECDC), Stockholm, Sweden

Dear reader of *Eurosurveillance*: If you have subscribed to receive our Table of contents or have an account on our site, you will soon receive an email asking you to confirm that you would like to continue receiving the weekly email alerts. Should you not have received such a request in the coming weeks, please check your spam filter. If we do not have a response from you within 30 days, we will unsubscribe you and remove all your personal details from the www.eurosurveillance.org site. If you change your mind, you can always sign up again using only your email address. We do not ask for any other personal data for those who subscribe only to the Table of contents newsletter.

Users with a full account on our site, which allows setting up personalised alerts and saved searches, will also receive the request to confirm if they want to retain their account. In line with our data protection policy, we will reduce the personal data stored in our website. For full accounts, we will continue to collect and store the following information: username, password, email address, and alert preferences set by the user. Other information you may have entered when you set up the account will be erased. 

Personal information collected by *Eurosurveillance* is processed under Regulation (EU) No 2018/1725 of the European Parliament and of the Council of 23 October 2018 on the protection of natural persons with regard to the processing of personal data by the Union institutions, bodies, offices and agencies and on the free movement of such data. Subscribers will be required to confirm their subscription every 3 years.

